# Construction of a Mammalian IRES-based Expression Vector to Amplify a Bispecific Antibody; Blinatumomab

**DOI:** 10.22037/ijpr.2019.14387.12351

**Published:** 2019

**Authors:** Fatemeh Naddafi, Fatemeh Davami, Maryam Tabarzad, Farzaneh Barkhordari, Farshad H. Shirazi

**Affiliations:** a *Pharmaceutical Sciences Research Center, Shahid Beheshti University of Medical Sciences, Tehran, Iran. *; b *Biotechnology Research Center, Pasteur Institute of Iran, Tehran, Iran. *; c *Protein Technology Research Center, Shahid Beheshti University of Medical Sciences, Tehran, Iran. *; d *Department of Toxicology, Shadid Beheshti University of Medical Sciences, Tehran, Iran.*

**Keywords:** FC550A-1 vector, Blinatumomab, phiC31 integrase, DHFR, CHO cells

## Abstract

Blinatumomab, the bispecific T cell engager antibody (BsAb), has been demonstrated as the most successful BsAb to date. Throughout the past decade, vector design has great importance for the expression of monoclonal antibody in Chinese hamster ovary (CHO) cells. It has been indicated that expression vectors based on the elongation factor-1 alpha (EF-1 alpha) gene and DHFR selection marker can be highly effective to produce populations of stably transfected cells in the selection medium. Moreover, the phiC31 integrase system is considered as an attractive and safe protein expression system in mammalian cells and it could integrate a donor plasmid of any size, as a single copy, in to the host genome with no cofactors. In this study, phiC31 integrase technology in combination with DHFR amplification system was used to have an expression vector for future expression of blinatumomab in CHO cells. The gene of interest (BsAb gene) could be joined to DHFR selection marker with the insertion of an internal ribosome entry site (IRES). By positioning the DHFR downstream of BsAb gene and IRES, the transcription of the selection marker can depend on the successful transcription of the BsAb gene, which was located upstream in the expression construct. In this study, FC550A-1 vector was used as the backbone and DHFR selection marker was successfully combined with phiC31 integrase technology to generate a high-expressing construct for BsAb expression in CHO-DG44 cells in future studies.

## Introduction

Sales of therapeutic proteins are expected to cross $125 billion by 2020 (1). More than 50% of approved therapeutic antibodies on the market, as the main therapeutic proteins, have been produced in mammalian cells, due to the ability of these cells to express proteins similar to naturally synthesized ones in the humans with the same molecular structures and biological functions (2). Chinese hamster ovary cells have been considered as the most common cells applied in the commercial production of therapeutic antibodies (3). It has been shown that the construct optimization for efficient transcription and translation of recombinant proteins can increase the copy number of foreign gene per cell. Consequently, higher expression levels can be achieved (4). Strong promoters such as the cellular elongation factor (EF) 1-alpha promoter and polyadenylation sites from the simian virus (SV) 40 have been used in expression constructs to improve mRNA stability and translation efficiency (5). phiC31 integrase, a site-specific recombinase from bacteriophage, is considered as a useful tool in mammalian cells. The enzyme mediates precise, unidirectional recombination between two attB and attP sites (6). 

Gene amplification could be an experimental strategy to increase protein production in mammalian cells (7). In the 1950s, dihydrofolate reductase (DHFR) gene-amplification mechanisms have been discovered and the original basis of recombinant protein production in dhfr^−^ CHO was formed (8). The DHFR system is well-known for high levels amplification and expression of recombinant genes (9). It has been indicated that expression constructs based on the elongation factor-1 alpha (EF-1 alpha) gene and DHFR selection marker might be used to have highly productive populations of stably transfected cells in the selection medium. This can be achieved by gradually increasing methotrexate (MTX) concentration, as the DHFR inhibitor, in the selection medium (10, 11). 

Bispecific T cell engager (BiTE) is considered the most common format for retargeting T cells to tumor cells in clinical studies (12). Blinatumomab, as the first-in-class BiTE against CD19 and CD3, was approved by the US Food and Drug Administration (FDA) on December 3, 2014 (13, 14). Blinatumomab has been proved to have promising results in patients with relapsed or refractory acute lymphoblastic leukemia (15). In this study, we focused on the combination of DHFR selection marker with phiC31 integrase technology to construct a high-expressing plasmid, which can be used in future studies to express Blinatumomab in CHO-DG44 cells. It is anticipated that this mammalian expression construct will amplify gene of interest, and also minimize random integration of the donor vector.

## Experimental

*Strains, plasmids and culture media*

The expression cassette was pcDNA3.1 (+) plasmid (Invitrogen). The sequence of Blinatumomab was sub-cloned from the pGH plasmid, into the pcDNA3.1 (+) plasmid by NheI/HindIII (Fermentas). Another expression cassette was FC550A-1 (System Biosciences), in which Blinatumomab gene was cloned with EcoRV restriction enzyme (Fermentas). IRES-DHFR gene was from pOptiVEC™-TOPO® plasmid (Invitrogen). *E.coli* strain Top10 F′ (Novagen) was used as the host for recombinant plasmid. *E. coli* was cultured in LB agar and Broth (Bio Basic).

*Amplification of DHFR-IRES gene with PCR*

IRES-DHFR gene was amplified by PCR using the following primers: IRES*-*DHFR*-*Fwd 5΄ataGAATTCTTAACGCCGCCCCTCTCC 3΄ and IRES-DHFR-Rev 5΄ttaGCGGCCGCGTTTTAGTCTTTCTTCTCGTAGACTTC 3΄. The underlined bases indicate ‎EcoRI and ‎NotI restriction sites. The PCR mixture consisted of 5 μL of 10 × PCR buffer, 2.5 mM MgCl2, 0.2 mM for each dNTP, 250 nM for each primer, 1 μL of template DNA, and 5 units of Pfu DNA polymerase (Fermentas) in the final volume of 50 μL. The amplification consisted of 35 cycles on a thermocycler (Eppendorf) as follows: preliminary denaturation for 5 min at 95 °C followed by 25 cycles including denaturation for 1 min at 95 °C, annealing for 1 min at 53 °C, extension for 1 min at 72 °C, and final extension for 10 min at 72 °C. PCR product fragment was electrophoresed on the 1% agarose gel and stained with ethidium bromide. After the PCR process, the amplified DNA fragments are size-separated by agarose gel electrophoresis and purified using the NucleoSpin^®^ Gel and PCR Clean-up kit (MN).

*Construction of the expression plasmid FC550A-1-BsAb-IRES-DHFR*

The BsAb gene was cloned into the expression plasmid pcDNA3.1 (+) with NheI and HindIII restriction enzymes. The PCR product and the pcDNA3.1 (+) plasmid were double-digested with the EcoRI and ‎NotI restriction endonucleases for 16h at 37 °C. Then, the digested fragments were electrophoresed on the 1% agarose gel. Subsequently, the fragments were purified using the NucleoSpin^®^ Gel and PCR Clean-up kit (MN) according to manufacturer′s instructions. Ligation was performed by using T4 DNA ligase enzyme (Fermentas). 100 ng of purified double-digested of cloned pcDNA3.1 (+) and 3-5 fold molar excess of insert (IRES-DHFR) were incubated with T4 DNA ligase enzyme and 10X T4 DNA ligase buffer at 22 °C for 4 h. The recombinant plasmid was transformed into the competent Top10 F′ by standard calcium chloride method. Transformants were selected on LB-agar medium containing Ampicillin (50 mg /mL). A single colony of *E. coli* cells carrying the ligated plasmid was grown in 5 mL LB medium containing Ampicillin (50 mg/mL). Plasmid extraction was performed using the FavorPrep Plasmid Extraction Mini Kit (FAPDE050- Favorgen Biotec Corp). Subsequently, the clones containing ligated plasmid were screened with XhoI and HindIII restriction enzymes. Then, the BsAb-IRES-DHFR gene was sub-cloned into the expression plasmid FC550A-1 by using CloneJET PCR Cloning Kit (K1231). Blunt-ended BsAb-IRES-DHFR gene generated with DNA Blunting Enzyme is ligated directly into the cloning plasmid (FC550A-1) which is digested with EcoRV. A single colony of *E. coli* cells carrying the desired gene (BsAb-IRES-DHFR) was cultured in 5 mL LB medium containing Ampicillin. Plasmid extraction was performed using the FavorPrep Plasmid Extraction Mini Kit (FAPDE050- Favorgen Biotec Corp). Subsequently, FC550A-1 containing BsAb-IRES-DHFR was evaluated with XhoI and SmaI (Fermentas) restriction enzymes.

## Results

*Construction of the expression plasmid FC550A-1-BsAb-IRES-DHFR*

**Figure 1:**
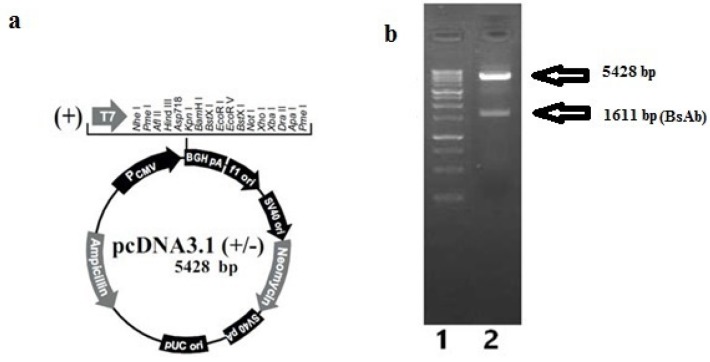
Confirmation of pcDNA 3.1 (+); a) annotated presentation of pcDNA3.1 (+); b) Gel electrophoresis on agarose 1%, lane 1: 1Kb DNA ladder (Fermentas), lane 2: digested pcDNA3.1 (+) plasmid

The BsAb gene was cloned successfully into the expression plasmid pcDNA3.1 (+) (Figure 1a) and confirmed by the digestion assay with NheI and HindIII restriction enzymes. Digestion of pcDNA3.1 (+) –BsAb plasmid resulted in two fragments, which can be detected as two distinct bonds on gel electrophoresis. The upper band was the backbone of the plasmid (5428 bp) and the lower was BsAb gene (1611 bp) (Figure 1b).

**Figure 2:**
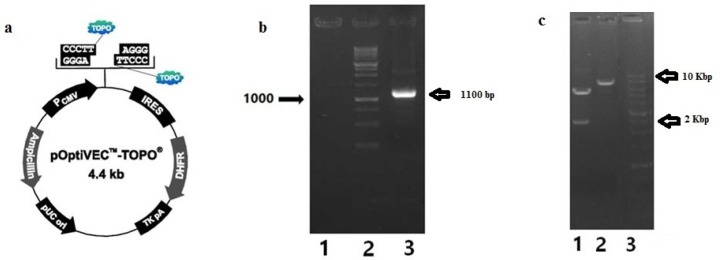
Amplification of IRES-DHFR gene from pOptiVEC™-TOPO® plasmid and cloning in to pcDNA 3.1(+); a) annotated presentation of pOptiVEC™-TOPO® plasmid; b) Gel electrophoresis on agarose 1% of amplified IRES-DHFR gene, lane 1: blank, lane 2: 1Kb DNA ladder (Fermentas), lane3: amplified IRES-DHFR gene (1100 bp); c) Gel electrophoresis on agarose 1% of digested pcDNA3.1 (+)-IRES-DHFR with XhoI and HindIII restriction enzymes, separately; Lane 1: pcDNA3.1 (+)-IRES-DHFR digested with XhoI (5652 bp and 2389 bp), Lane 2: Digested pcDNA3.1 (+)-IRES-DHFR with HindIII (~8000 bp), lane 3: 1Kb DNA ladder (Fermentas)

IRES-DHFR gene was amplified by IRES-DHFR-Fwd and IRES-DHFR-Rev primers, containing restriction sites, from pOptiVEC™-TOPO® plasmid (figure 2a). EcoRI and ‎NotI restriction sites were introduced at the 5´ and 3´ of the IRES-DHFR gene, respectively. Successful amplification of 1100 bp of IRES-DHFR gene was visualized on 1% Agarose (Figure 2b). Then, IRES-DHFR gene was cloned successfully into the pcDNA3.1 (+)-BsAb plasmid. The pcDNA3.1 (+)-BsAb-IRES-DHFR was digested with XhoI and HindIII restriction enzymes. Digestion of pcDNA3.1 (+)-BsAb-IRES-DHFR plasmid with XhoI led to two detectable bonds on agarose gel electrophoresis. The upper band was 5652 bp and lower was 2389 bp. Digestion of pcDNA3.1 (+)-BsAb-IRES-DHFR with HindIII resulted in the linear vector (Figure 2c).

**Figure 3:**
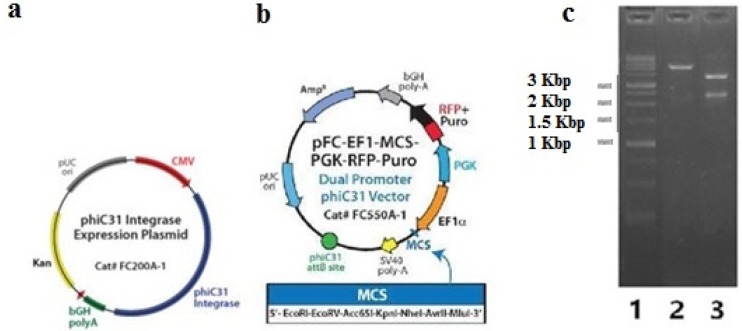
Confirmation of FC550A-1- BsAb-IRES-DHFR; a) annotated presentation of FC200A-1 as phiC31 integrase expression vector that was co-transfected with FC550A-1- BsAb-IRES-DHFR; b) annotated presentation of FC550A-1; c) Gel electrophoresis on agarose 1% of digested FC550A-1- BsAb-IRES-DHFR construct with XhoI and SmaI restriction enzymes, lane a: 1Kbp ladder (Fermentas), lane2: digested FC550A-1- BsAb-IRES-DHFR with SmaI resulted in linear construct, lane 3: digested FC550A-1- BsAb- IRES-DHFR with XhoI resulted in three fragments that were 3401 bp, 2385 and 2975 bp

Then, the BsAb-IRES-DHFR gene was sub-cloned into the expression plasmid, FC550A-1. Subsequently, the clones containing FC550A-1- BsAb-IRES-DHFR construct were screened with XhoI and ‎SmaI restriction enzymes. The digestion of FC550A-1 with SmaI made the linear form of construct, which indicated that the gene of BsAb-IRES-DHFR has cloned in the vector, and digestion with XhoI resulted in three fragments that were 3401 bp, 2385, and 2975 bp, which again confirmed the presence of BsAb-IRES-DHFR gene in the final construct (Figure 3). 

The results confirmed that BsAb-IRES-DHFR gene was successfully cloned into the expression vector, FC550A-1.

**Figure 4:**
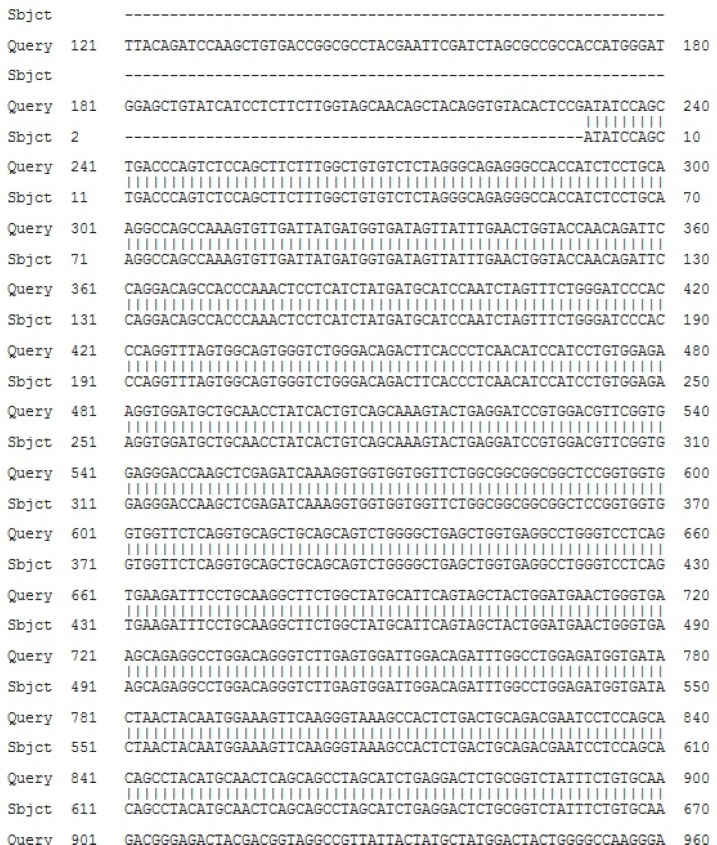
Sequence analysis of FC550A-1- BsAb-IRES-DHFR construct; Gene sequence of BsAb-IRES-DHFR was aligned with the sequence of FC550A-1- BsAb-IRES-DHFR by global alignment of Blast (https://blast.ncbi.nlm.nih.gov/Blast.cgi)

Moreover, the sequence analysis of recombinant FC550A-1-BsAb**-**IRES-DHFR plasmid confirmed that there were no amplification errors (Figure 4) and BsAb-IRES-DHFR gene was completely cloned in the correct direction.

## Discussion

It has been demonstrated that high-level recombinant protein expression could be achieved by amplification of the gene of interest with a selectable marker, such as DHFR. DHFR gene amplification in DHFR^－^ CHO cells would result in amplification of the co-linked target gene (16). In another study, the monoclonal antibody gene was successfully cloned into both pcDNA™ 3.3-TOPO® and pOptiVEC™ TOPO® shuttle vectors, separately, in order to investigate the role of DHFR amplification system in CHO (17). However, in this study, we decided to construct one expression cassette to have DHFR amplification system, which would be cloned by using phiC31 integrase technology, in order to achieve higher expression level of this recombinant protein. FC550A-1 plasmid, as the cloning vector bearing BsAb-IRES-DHFR gene, was co-transfected with FC200A-1 plasmid (phiC31 Integrase Expression Plasmid), and this co-transfection led to the integration of cloning vector into pseudo-attP sites in the CHO genome through the attB × attP recombination. We used this cloning method to have both DHFR selection marker with phiC31 integrase technology in one expression system. IRES has been used to construct bi- or oligo-cistronic expression vectors, to co-express different genes from one mRNA. Moreover, a study reported that using this technology could result in the overexpression of the proteins in CHO cells (18). In another study, the gene which is positioned upstream of the IRES sequence, was expressed at higher level as compared with the gene downstream of the IRES (19-23). Therefore, we constructed an expression vector containing an IRES followed by a selectable marker (DHFR) and the gene of interest (BsAb) is placed at the 5′ site of IRES sequence under the control of the Elongation factor 1-alpha promoter. The final construct was checked by enzymatic digestion and sequencing to ensure the right orientation, and DNA sequence.

## Conclusion

Due to the increasing demand for therapeutic monoclonal antibodies, there has been a lot of interest in achieving maximum production of these biopharmaceuticals. Current study was based on a recently developed gene amplification technology and phiC31 integrase-mediated site-specific recombination. PhiC31 integrase-based vector, FC550A-1, as the backbone. The BsAb-IRES-DHFR gene was successfully cloned into FC550A-1 expression vector and now, is ready for future expression of bispecific antibody in CHO-DG44 cells. Similar approach can be applied to achieve effective expression construct for other monoclonal antibodies in CHO- DG44 stable expression system. Moreover, by inclusion of a selectable marker (DHFR) in the mammalian expression vector, CHO cells will be derived with stable gene amplification. 

## References

[1] Ecker DM, Jones SD, Levine HL (2015). The therapeutic monoclonal antibody market. MAbs.

[2] Zhu J (2012). Mammalian cell protein expression for biopharmaceutical production. Biotechnol. Adv.

[3] Lifely MR, Hale C, Boyce S, Keen MJ, Phillips J (1995). Glycosylation and biological activity of CAMPATH-1H expressed in different cell lines and grown under different culture conditions. Glycobiology.

[4] Bendig M (1988). The production of foreign proteins in mammalian cells. Genet. Eng..

[5] Nott A, Le Hir H, Moore MJ (2004). Splicing enhances translation in mammalian cells: an additional function of the exon junction complex. Gene. Dev..

[6] Keravala A, Calos MP (2008). Site-specific chromosomal integration mediated by phiC31 integrase. Methods Mol. Biol.

[7] Kellems RE (1991). Gene amplification in mammalian cells: strategies for protein production. Curr. Opin. Biotech.

[8] Agrawal V, Bal M (2012). Strategies for rapid production of therapeutic proteins in mammalian cells. BioProcess Int.

[9] Kingston RE, Kaufman RJ, Bebbington C, Rolfe M (2002). Amplification using CHO cell expression vectors. Curr. Protocol. Mol. Biolog.

[10] Deer JR, Allison DS (2004). High‐Level Expression of Proteins in Mammalian Cells Using Transcription Regulatory Sequences from the Chinese Hamster EF‐1α Gene. Biotechnol. Prog.

[11] Orlova NA, Kovnir SV, Hodak JA, Vorobiev II, Gabibov AG, Skryabin KG (2014). Improved elongation factor-1 alpha-based vectors for stable high-level expression of heterologous proteins in Chinese hamster ovary cells. BMC. Biotechnol.

[12] Spiess C, Zhai Q, Carter PJ (2015). Alternative molecular formats and therapeutic applications for bispecific antibodies. Mol. Immunol.

[13] Wu J, Fu J, Zhang M, Liu D (2015). Blinatumomab: a bispecific T cell engager (BiTE) antibody against CD19/CD3 for refractory acute lymphoid leukemia. J. Hematol. Oncol.

[14] Naddafi F, Davami F (2015). Anti-CD19 monoclonal antibodies: a new approach to lymphoma therapy. Int. J. Mol. Cell. Med.

[15] Buie LW, Pecoraro JJ, Horvat TZ, Daley RJ (2015). Blinatumomab: a first-in-class bispecific T-cell engager for precursor B-cell acute lymphoblastic leukemia. Ann. pharmacother.

[16] Akbarzadeh-Sharbaf S, Yakhchali B, Minuchehr Z, Shokrgozar MA, Zeinali S (2013). Expression enhancement in trastuzumab therapeutic monoclonal antibody production using genomic amplification with methotrexate. Avicenna J. Med. Biotechnol.

[17] Akbarzadeh-Sharbaf S, Yakhchali B, Minuchehr Z, Shokrgozar MA, Zeinali S (2012). In silico design, construction and cloning of Trastuzumab humanized monoclonal antibody: a possible biosimilar for Herceptin. Adv. Biomed. Res.

[18] Hennecke M, Kwissa M, Metzger K, Oumard A, Kröger A, Schirmbeck R, Reimann J, Hauser H (2001). Composition and arrangement of genes define the strength of IRES-driven translation in bicistronic mRNAs. Nucleic. Acids Res.

[19] Kaufman RJ, Davies MV, Wasley LC, Michnick D (1991). Improved vectors for stable expression of foreign genes in mammalian cells by use of the untranslated leader sequence from EMC virus. Nucleic. Acids Res.

[20] Houdebine LM, Attal J (1999). Internal ribosome entry sites (IRESs): reality and use. Transgenic Res.

[21] Dirks W, Wirth M, Hauser H (1993). Dicistronic transcription units for gene expression in mammalian cells. Gene.

[22] Zhou Y, Aran J, Gottesman MM, Pastan I (1998). Co-expression of human adenosine deaminase and multidrug resistance using a bicistronic retroviral vector. Hum. Gene Ther..

[23] Bouabe H, Fässler R, Heesemann J (2008). Improvement of reporter activity by IRES-mediated polycistronic reporter system. Nucleic Acids Res.

